# A Discussion on Hospital Construction

**Published:** 1887-11-26

**Authors:** 


### THE HOPITALS ASSOCIATION.

A DISCUSSION ON HOSPITAL CONSTRUCTION.

(*Continued from page* 139.)

DR. CLIFFORD BEALE (Great Northern Central Hospital: There is a point to which I should like to draw attention. One great disadvantage common to several of the London hospitals is the prevalence of draught. Many cases which ought to do well, at a critical time get exposed to draughts, catch cold, and do badly. If any system could be devised by which a ward could be well ventilated without draughts it would be one of the greatest things an architect could achieve.

Mr. Collins, F.R.I.B.A.: Let the architect arrange his buildings as he may, the best scheme—well-devised and thoughtfully planned—will eventually and certainly fail if those who are subsequently entrusted with its care do not properly carry out what the architect has intended. Do not blame the architect but those who have the care of such institutions. I do not find the same fault with regard to ventilation by means of tubes as previous speakers have done, but of course if tubes are not kept clean bad results must follow. In regard to corridors, I may say that at the Metropolitan Free Hospital I have taken great care to keep them entirely bathed in and surrounded by air. We have had to build with extreme economy, and to construct storey upon storey, making what I believe Dr. Richardson terms a "storehouse of disease." The hospital has five wards altogether, each containing twenty-four beds. In the erection of this hospital I have, as I have said, been cramped by questions of economy, and hence the arrangements have had to be as simple and inexpensive as possible. A rather witty observation was made at the annual dinner by the Hon. Mr. Yorke, who said he had gone over the hospital, and thought, from its simplicity of construction, no architect had been employed at all! (Laughter.) At the hospital I have designed we have had to practise the most rigid economy. The walls we have covered with Portland cement, and painted them over with silicate paint, which has given them a nice enamelled surface. With regard to underground communication, I think that, if subterranean passages are kept in proper order and condition, there is no objection to them; on the contrary, there are some great advantages. Adequate provision does not seem to have been made for fire escape. I certainly think it worth while to consider whether, by means of corridors or otherwise, some plan could be devised for the escape of patients from the upper wards of hospitals.

Mr. Fardon (Medical Superintendent, Middlesex Hospital): Mr. Young has certainly covered in his paper a very wide area. With regard to the construction of hospitals on the separate pavilion principle, I cannot claim to have any practical experience, but I have been rather struck with one fact, and that is, that all those gentlemen who have advocated this principle for the construction of hospitals have failed to push their arguments home by proving that a better result is attained by separate pavilions than in hospitals constructed storey upon storey. In London, where ground is so expensive, but where it is at the same time essential that we should have our hospitals—for we are obliged to build our hospitals in the centres of large populations—it is well-nigh impossible to construct hospitals upon the separate pavilion principle; and, therefore, Mr. Young has, I think, done wisely in endeavouring to show us how he can construct a hospital storey upon storey, and at the same time one eminently satisfactory in its results. If you have hospitals built in separate pavilions the cost of administration will be most enormously increased, besides which there are other disadvantages, such as that of taking hot diet from the kitchen along exposed corridors. It is also quite impossible to keep underground passages in the state in which they ought to be kept. If you put obstacles in the way of quickly conveying food and clean linen to the patients, and of removing foul linen, of quickly summoning medical assistance, etc., you will, I say, interfere with the welfare of your patients. This is a matter which should be earnestly considered. With regard to the question of ventilation, I have had no experience whatever, except in the ventilation of this hospital, the Middlesex. Here we have no artificial system at all. It would, I think, be an advantage if there were some system of ventilation which was not entirely under the control of the nurses, etc., who administered the ward. Patients, who do not understand fully the value of fresh air, often remonstrate at the least breath, and it would at times be very difficult to contend against their objections if the superintendent nurses had complete control and windows were surreptitiously closed. That would lead to the ventilation of wards by the opening of doors, which is extremely deleterious, because this would merely ventilate the wards from the corridors, themselves ventilated from other wards. I think the danger of exposing patients who are lying in bed to draughts is much exaggerated, and may be provided against by a simple arrangement of curtains.

Mr. Osborne Smith: It is all very well to have artificial means of ventilation, but you generally find that things which need control are not controlled in a proper manner, and are then worse than useless. If warm air is admitted in such a way as not to produce draughts, and with plenty of shafts to take away foul air, a tolerably pure atmosphere can be maintained by simple means of natural ventilation without tubes to be clogged with dust and dirt. I fully endorse the views which one speaker has expressed with reference to patients being afraid of air. Some of them are afraid of the air touching them, and fancy draughts injure them where draughts do not exist. I think it far more likely that effective ventilation can be accomplished by natural ventilation than by any complicated system of the artificial introduction of air, and I am therefore exceedingly pleased to notice so much emphasis has been put on the system of natural ventilation, which is, I believe, the only system by which such buildings as hospitals can be effectively and satisfactorily ventilated.

Mr. Thomas Ryan remarked that not very long since hospitals were, in many cases, designed without, as it were, any regard for the purposes they were to fulfil, and he thought that a debt of gratitude was due to the Hospitals Association for having brought about that discussion that evening, and for any good that was likely to come of it. Hospitals should suit the purposes for which they were intended. Referring to the connexion between hospital construction and management, he pointed out that small wards multiplied the number of nurses necessary, and therefore increased the cost of management. He was entirely in favour of natural ventilation by window, and he thought exaggerated importance was sometimes given to the question of draughts. Sisters and nurses could arrange screens, and by these means the question of draughts could be met.

Mr. Stainton expressed his opinion that the staircases and corridors of hospitals should be heated to a temperature slightly above that of the wards themselves. He thought also that foul air should be burnt before being passed out, a method which would be of especial value in the case of fever hospitals.

Mr. Brydon asked what value Mr. Young attached to inspection windows.

Mr. Spencer Watson: It has occurred to me that architects have not laid down for themselves any principle by which they can guide themselves as to the success of their labours. I should like to know whether Mr. Young is prepared to lay down any index to success. There have been in some hospitals outbreaks of pyæmia, erysipelas, etc., which have pointed to some failure of construction. In constructing hospitals architects occasionally lose sight of the fact that economy is a very serious point, because it is very difficult for those who manage hospitals to raise the money for the purpose of carrying out the views of the architects, and therefore I do not think they should attempt to do too much. In the construction of the new hospital at Holloway—the Great Northern Central Hospital—we have had to contend with a difficulty of this kind. The architect, for instance, proposed a very expensive kind of floor—solid block floor—which was doubtless very perfect, but more fit for a palace than a hospital. In building a hospital we should not be led into mistakes of that kind. A block floor was not necessarily the most advisable, even from a sanitary point of view.

Dr. Potter thought the question of draughts was of the greatest importance. He did not wish to be uncivil, but did some architects make light of draughts because they could not build wards draughtless?

Mr. Young, in replying to the points raised in the discussion, said: I am not surprised that the discussion has rather tended to gravitate towards the question of ventilation because that is such an exceedingly pleasant subject; everyone has his or her own ideas on this question of draughts. One doctor said it was of little or no consequence. Well, I have arranged some huts to meet the present fever epidemic, and the medical officer of the institution to which I refer, in discussing the question of keeping the patients warm, said that the question of draughts was simply the question of another blanket or two on the patient, adding, "Let us have all the air we can through the huts." Very considerable stress was laid on this question in a paper by Mr. Holmes, and Sir Andrew Clark said it was something scandalous the amount of draughts felt in hospital wards. This conflict of opinion puts us poor architects in a difficult position; we cannot comply with both requirements, draughts and draughtless wards too. It seems to me that the balance of evidence is in favour of doing one's utmost to prevent draughts; and that, I think, we have done at the Great Northern. There is no originality about the mode of window there. It was used a number of years ago for schools, but whether its application for hospital wards is original or not I cannot say. Now, as to the question raised by Mr. Spencer Watson regarding the index of success, I wish some doctor would tell us what is to be the index of success. It cannot be for an architect to work out for himself any test or comparison between one hospital and another by the results of septic disease or any sick tables of the kind. If we could arrive at some definite index of comparative success it would be of very great value, but I cannot see how it can be arrived at by architects, or how they can originate it. With regard to inspection-windows, all I can say is that I have never found yet in all the hospitals I have been to that an inspection window was made any use of whatever. The usual aspect of an inspection window is an opaque muslin blind, a pile of books, and on those a pot of flowers. If anyone could see through that I should be surprised (laughter). I have never heard that there was any value whatever in having inspection windows.

General Keatinge: I would very willingly have said a few words from an administrative point of view as to the extent to which this so-called pavilion system may be carried, so that we might know how many miles a nurse, etc., might be expected to walk in a day. But I will refrain on the present occasion, and will say nothing, but propose a vote of thanks to the Weekly Board of this hospital for the use of this room, to the Secretary (Mr. Bartholeyns), and also to Sir Douglas Galton for presiding (cheers.)

Mr. Burdett, in seconding the vote of thanks, said, I think our thanks are cordially due to Sir Douglas Galton, and I hope he will agree with me that this meeting, which has been called to listen to architects, has been one which has been attended with success, a success which warrants a frequent repetition for the benefit of architects, of hospital administrators and of the public generally, especially of the sick members of the public. I hope as the fruit of such meetings as this that the Hospitals Association may constitute a sectional committee called the Construction and Building Committee, which shall assist hospital administrators and architects in conferring and keeping note of all these features of hospital construction and publish from time to time their notes on hospital construction. I have also to express on behalf of the Council of the Hospitals Association our thanks to the authorities of the Middlesex Hospital for the use of this board room. This meeting at hospitals is a new departure, and one which will, I think, commend itself to all interested in the welfare of these grand institutions. The Council of this Association desire that their meetings shall be held in the different board rooms of the hospitals throughout London, and possibly our Chairman will co-operate to see if we cannot have a meeting in one of the hospitals of the Metropolitan Asylums Board. We shall always be glad of offers of this kind. The next meeting will be held, by kind permission of the Governors, in the court room of Guy's Hospital, and will be presided over by the Treasurer, when a paper will be read by Dr. Steele on the "Registration of Nurses."

The resolution of thanks having been unanimously voted, and the Chairman with Dr. Coupland briefly responded, and the meeting then terminated.

